# No Evidence for Orthohepevirus C in Archived Human Samples in Germany, 2000–2020

**DOI:** 10.3390/v14040742

**Published:** 2022-03-31

**Authors:** Mirko Faber, Jürgen J. Wenzel, Monika Erl, Klaus Stark, Mathias Schemmerer

**Affiliations:** 1 Department for Infectious Disease Epidemiology, Robert Koch Institute, 10437 Berlin, Germany; starkk@rki.de; 2 National Consultant Laboratory for HAV and HEV, Institute of Clinical Microbiology and Hygiene, University Medical Center Regensburg, 93053 Regensburg, Germany; juergen.wenzel@klinik.uni-regensburg.de (J.J.W.); monika.erl@klinik.uni-regensburg.de (M.E.)

**Keywords:** hepatitis E virus, orthohepevirus C, rat HEV, epidemiology, phylogeny, Germany

## Abstract

*Orthohepevirus C1*, also known as rat hepatitis E virus (HEV), has been shown to sporadically cause disease in immunocompromised and immunocompetent adults. While routine serological assays vary in reactivity, rat HEV is not detected in routine HEV RT-PCR. Thus, such infections could be either missed or misclassified as conventional HEV (*Orthohepevirus A*) infections. We conducted a retrospective screening study among serum and plasma samples from patients suspected of having HEV infection, which were archived at the national consultant laboratory for HAV and HEV between 2000 and 2020. We randomly selected *n* = 200 samples, which were initially tested reactive (positive or borderline) for HEV-IgM and negative for HEV RNA and re-examined them using a highly sensitive *Orthohepevirus C* genotype 1-specific in-house RT-qPCR (LoD 95: 6.73 copies per reaction) and a nested RT-PCR broadly reactive for *Orthohepevirus A* and *C*. Conventional sanger sequencing was conducted for resulting PCR products. No atypical HEV strains were detected (0 of 200 [0.0%; 95% confidence interval: 0.0%–1.89%], indicating that *Orthohepevirus C* infections in the investigated population (persons with clinical suspicion of hepatitis E and positive HEV-IgM) are very rare.

## 1. Introduction

*Orthohepevirus A* (hepatitis E virus, HEV-A), a member of the *Hepeviridae* family, is endemic worldwide causing sporadic cases and outbreaks of acute hepatitis. Depending on the genotype, it is typically spread human to human via the fecal–oral route (genotypes 1 and 2) or zoonotically through consumption of animal products, particularly pork (genotypes 3 and 4) [1]. Other HEV-A genotypes (5, 6, 7 and 8) have been detected in wild boar and camelids in Asia with only one human case described so far [2].

Additional members of the *Orthohepevirus* genus (species B, C and D) circulate in birds, rodents, ferrets and bats [3]. *Orthohepevirus C* is divided into 2 genotypes. Genotype 1 (“rat HEV”, HEV-C1) was first isolated from brown rats in Germany in 2010 [4] and subsequently shown to widely circulate among multiple species of rat on at least three continents [5]. Genotype 2 (HEV-C2) was found in species belonging to the order of Carnivora [6]. Due to a large phylogenetic distance to HEV and its inability to experimentally infect pigs or nonhuman primates, HEV-C was assumed to not be a source of human infections [7,8].

It has only recently been shown that HEV-C1 is able to cause disease in immunocompromised and immunocompetent adults [9,10]. Sporadic cases of NAT-confirmed rat HEV infections were so far reported from Hong Kong, Spain and in one Canadian individual with travel history to the African continent [9,10,11,12,13].

Commercial serological assays have shown variable sensitivity (between 10% and 70%) to *Orthohepevirus* non-A strain infections [14]. However, this is not the case for NATs used in routine diagnostics, which are designed to detect conventional genotype 1 to 8 HEV-A. Thus, infections with atypical HEV strains could frequently be either missed or misclassified as conventional HEV infections when molecular typing was unsuccessful [IgM(+)/PCR(−)].

Information on the incidence of such infections in the population is scarce. So far, Sridhar et al. reported that HEV-C1 infections accounted for 8% of all genotyped hepatitis E cases in Hong Kong and concluded that the absence of HEV RNA in serum testing positive for HEV-IgM antibodies should trigger testing for HEV-C1 [11]. The screening of various patient populations in southwest Hungary and southwestern France showed no evidence of HEV-C1 circulation [15,16], while from Spain a prevalence of 2.5% (95% CI: 0.06–13.1) was reported for a cohort of patients diagnosed with acute Hepatitis E [13].

The objective of this study was to estimate the prevalence of HEV-C1 infections among persons with a clinical suspicion and laboratory evidence of hepatitis E infection in Germany.

## 2. Materials and Methods

We conducted a retrospective screening study among a random selection of *n* = 200 archived serum and plasma samples referred to the German consultant laboratory for HAV and HEV. The patients’ specimens were referred for further investigation of a clinical suspicion of hepatitis E (e.g., jaundice, upper abdominal pain or elevated liver enzymes) in the years 2000 through 2020. They typically consisted of residual serum or plasma taken for primary serological diagnostics in the acute phase. All specimens had been stored at −20°C, typically without additional freeze-thaw cycles. We selected a subset of specimens that had tested reactive (positive or borderline) for HEV-IgM using commercially available serological assays (Mikrogen *recom*Well and/or *recom*Line HEV) and negative using standard HEV-RT-qPCRs [17,18,19]. The sample size was chosen assuming there was a 2% prevalence of Orthohepevirus C genotype 1 in our sample which would, based on the normal approximation, allow to estimate the prevalence in the population (individuals with clinical suspicion of HEV infection with detectable HEV-IgM and no detectable HEV RNA) between 0 and 4% with 95% confidence.

We re-evaluated each of the 200 selected archived specimens using two different PCR reactions:

1. a nested RT-PCR broadly reactive for *Orthohepevirus A* and *C* as described in [4] and
2. an *Orthohepevirus C* genotype 1 specific RT-qPCR that was specifically established for this study.

**Figure 1:**
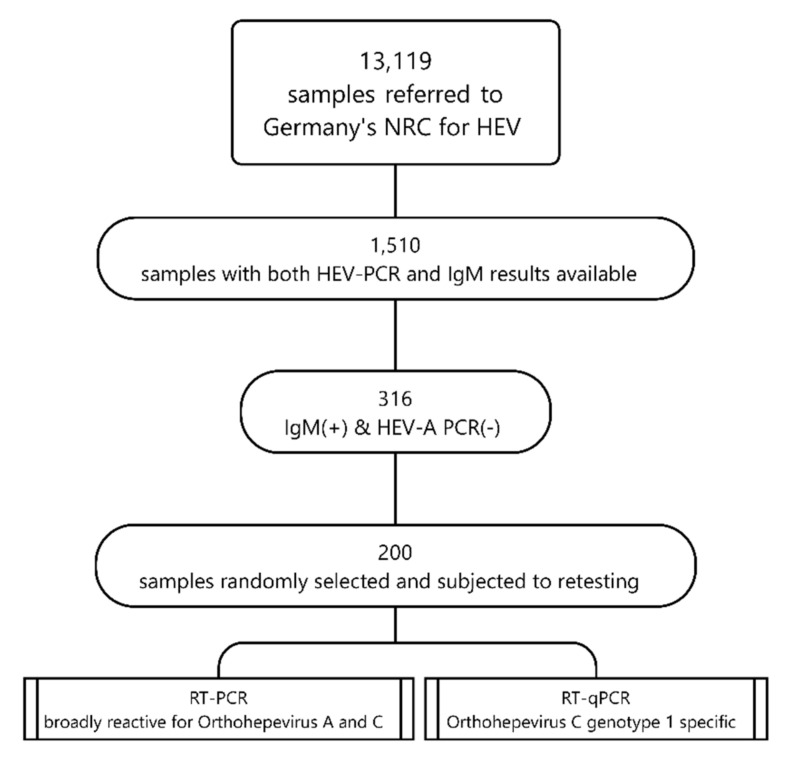
Sampling and testing algorithm applied in the retrospective screening study, Germany, 2000–2020.

Figure 1 summarizes the sampling and testing algorithm applied in this study.

**Figure 2:**
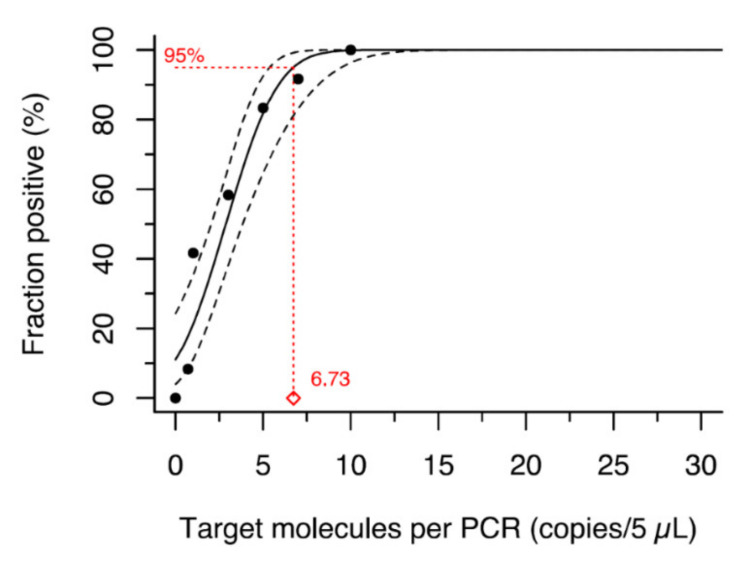
Limit of detection (LoD) 95 of HEV-C1 specific PCR based on strain R63/DEU/2009 plasmid as determined by probit analysis.

As for the newly established *Orthohepevirus C* genotype 1 specific RT-qPCR, primer and probe sequences are shown in Table 1. Optimal oligonucleotide concentrations were determined in titration experiments to maximize analytical sensitivity. The final composition of each 30 µL reaction was 15.7 µL DEPC-treated water, 7.5 µL TaqPath™ 1-Step RT-qPCR master mix (ThermoFisher Scientific, Waltham, MA, USA), 0.75 µL of 10 µM probe (end concentration 250 nM), 0.15 µL of 10 µM forward primer (end concentration 50 nM), 0.90 µL of 10 µM reverse primer (end concentration 300 nM) and 5 µL of template RNA. The procedure started with a reverse transcription at 50 °C for 15 min followed by an enzyme activation step at 95 °C for 2 min, 45 PCR cycles at 95 °C for 5 s and 60 °C for 30 s, and a final cool down to 4 °C. A plasmid based on the HEV-C1 strain R63/DEU/2009 served as a standard for absolute quantification [20]. The PCR’s LoD 95 was estimated by probit analysis at 6.73 copies per reaction (95% CI: 5.37–9.46; Figure 2). Nucleic acid preparations from two HEV-C1 positive cell culture supernatants (passage of strain R63 in PLC/PRF/5 and HuH-7-Lunet BLR cells; strain described in [20]) were used in each PCR run as positive controls.

PCR products (amplified by the broadly reactive nested PCR) were purified and sequenced using conventional Sanger sequencing on an ABI 3130xl automated sequencer. The resulting electropherograms were analyzed and assembled with CodonCode Aligner v4.2.7 (www.codoncode.com, CodonCode Corporation, Centerville, MA, USA). Consensus sequences were genotyped using the latest HEV reference set [21] extended with NCBI *Hepeviridae* reference sequences (GenBank accessions NC_015521.1, NC_018382.1, NC_023425.1, NC_038504.1, NC_040835.1 and NC_040710.1) as database for fasta36 similarity searches [22]. Sequence data from this article have been deposited with the International Nucleotide Sequence Database Collaboration Libraries (GenBank, DDBJ and ENA) under accessions numbers OK493454–7.

Statistical data analysis was conducted using STATA 17 (StataCorp, College Station, TX, USA). Confidence intervals were calculated using the Wilson score interval.

## 3. Results

Between 2000 and 2020, *n* = 13,119 specimens from 10,121 patients had been referred to the German consultant laboratory for HAV and HEV for a clinical or laboratory suspicion of HEV infection. Of these patients, *n* = 6200 (61.3%) and *n* = 5431 (53.7%) had been tested for the presence of HEV RNA and IgM. Both HEV PCR and IgM testing was conducted in *n* = 1510 patients, of which *n* = 316 (20.9%) were IgM(+)/PCR(-), fulfilling the selection criterion. Table 2 shows the summarized demographic patient data of the 200 randomly selected specimens that underwent further analysis. The distribution of anti-HEV IgM titers was as follows: borderline: 7; low positive: 33; mid positive: 31; high positive: 6 for samples tested using Mikrogen *recom*Well. The Mikrogen *recom*Line HEV differentiates only borderline (*n* = 27) and positive (*n* = 106).

In the broadly reactive nested RT-PCR, a product was detected in 4 of 200 (2.0%) specimens. Sanger sequencing revealed typical HEV genotypes associated with pig or wild boar (i.e., *Orthohepevirus A* genotype 3a (*n* = 1) and 3f (*n* = 3), GenBank accessions OK493454–7). Demographics of the respective patients were compatible with demographics of patients with conventional genotype 3 HEV infection in Germany (age range: 31–72 years, 2 male, 2 female). In the HEV-C1-specific RT-qPCR, all specimens tested negative for HEV-C1 RNA. Calculating Wilson’s score interval reveals a 95% confidence interval of 0.0% to 1.89% around the proportion of 0/200 (0.0%) *Orthohepevirus C* positive specimens.

## 4. Discussion

In this study, we re-investigated archived specimens of patients with a clinical suspicion and laboratory evidence of hepatitis E regarding the presence of HEV-C (including “rat HEV”) infection in Germany. Among 200 specimens investigated by a nested RT-PCR broadly reactive for Orthohepevirus A and C and a newly developed Orthohepevirus C genotype 1 specific RT-qPCR, we found no evidence of human HEV-C infection.

This result was rather unexpected given that HEV-C1 was reported to account for 8% of all genotyped hepatitis E cases in Hong Kong [14], the presence of the virus in the rodent reservoir in Germany [23] and a previous report on indirect (serological) evidence of past HEV-C1 infection in a cohort of forestry workers in Germany [24]. However, it is in line with studies conducted in southwest Hungary and southwestern France that investigated in/out-patients with hepatitis of unknown origin and predominantly immunosuppressed patients who tested negative for HEV-A RNA, respectively [15,16]. Both studies focused on patient populations with a high chance of discovering HEV-C1 cases but found none. Taken together, this might indicate that HEV-C1 does not circulate among the investigated populations or is much rarer in Europe than expected or compared to Hong Kong.

HEV-C1 infections, however, do occur in Europe, as shown in a very recent publication from Spain. Rivero-Juarez et al. reported three cases, two among 169 patients with hepatitis of unknown etiology (1.18%) and one additional case among 40 IgM-positive and HEV-A-RNA-negative hepatitis E patients (2.5%) [13]. The reason for these potential differences in prevalence between countries is unclear, as are transmission routes and risk factors for human HEV-C1 infection. None of the cases from Spain and Canada, and only a minority of the cases from Hong Kong, reported contact to rats or seeing rat droppings before infection. A case control-study on risk factors for acute hepatitis E in Germany showed the frequency of contact to rodents was not increased among cases compared to individually matched controls [25]. Thus, other transmission routes, e.g., through contaminated food items, should at least be considered.

Based on finding zero HEV-C positive among 200 specimens and the corresponding 95%-CI, the prevalence in a population with hepatitis E compatible symptoms and serological evidence of HEV infection (IgM) in Germany are estimated to be below 2%. This should be seen as a conservative estimate because of our additional selection criteria (negative HEV-A specific PCR), which increased the probability to find HEV-C1 in our sample. Thus, the actual proportion is—if positive at all—likely to be even lower.

Our study has some limitations: First, there is no information as to the health status and living conditions of the individuals tested in our study. While rat HEV has been detected in immunocompetent and immunocompromised individuals, the prevalence appears to be higher in immunocompromised individuals [11]. Additional studies in risk groups (e.g., immunosuppressed persons or those with frequent and close contact to rats) might thus be warranted. Second, we cannot completely rule out viral RNA degradation for samples stored at −20 °C for up to 19 years. Since we detected HEV-A RNA in a sample stored for 16 years, we assume that degradation likely plays a minor role. Third, the HEV-C sensitivity of the serological assays used in this study (Mikrogen *recom*Line and *recom*Well HEV-IgM) is unknown. While serology constitutes no ideal screening method for HEV-C1 infections, its application is expected to enrich the population with regard to the probability of finding HEV-C1 to some degree at least.

Based on our findings, we cannot exclude that sporadic hepatitis E cases related to *Orthohepevirus* C genotype 1 strains (such as those found in domestic and brown rats) do occur in Germany. However, their role in the current epidemiology of hepatitis E in Germany is likely very small, particularly compared to the frequency of HEV-A genotype 3 infections [26] and corresponding incident cases of disease [27].

Nonetheless, we recommend rat HEV infections to be considered in the extended differential diagnosis of viral hepatitis. In patients with a high clinical suspicion, risk factors (e.g., contact to rodents) and inconclusive laboratory results, second-line testing by broad-range NAT also covering rat HEV should be initiated.

## Figures and Tables

**Table 1:** Primers and probe sequences used for Orthohepevirus C genotype 1 (HEV-C1, rat HEV) RT-qPCR detection.

Name	Sequence	Location at NC_038504
HEV-C1_fwd	5′–TACTGCTAGAGAGGCCCAG–3′	49–67
HEV-C1_rvs	5′–GCTGTATCGGATGCGACC–3′	198–215
HEV-C1_prb	5′–6-FAM-ACCGCCTTTGCTAATGCT-NFQ-MGB–3′	86–103

Abbreviations: 6-FAM, 6-Carboxyfluorescein; fwd, forward primer; MGB, minor groove binder; NFQ, nonfluorescent quencher; prb, probe; rvs, reverse primer.

**Table 2:** Study population by demographic characteristics, Germany, 2000–2020 (*n* = 200).

Characteristic	*n*	%
**Sex**		
Female	98	49
Male	94	47
Unknown	8	4
**Age group**		
≤9	3	1.5
10–19	24	12
20–29	24	12
30–39	24	12
40–49	43	21.5
50–59	30	15
60–69	23	11.5
70–79	20	10
≥80	1	0.5
Unknown	8	4
**Place of residence**		
North	26	13
West	48	24
East	25	12.5
South	100	50
Unknown	1	0.5
**Total**	**200**	**100**
